# Genotypic and Phenotypic Characterization of *Staphylococcus aureus* Isolates from the Respiratory Tract in Mechanically-Ventilated Patients

**DOI:** 10.3390/toxins13020122

**Published:** 2021-02-06

**Authors:** Alicia Lacoma, Maisem Laabei, Jose Francisco Sánchez-Herrero, Bernadette Young, Gerard Godoy-Tena, Meissiner Gomes-Fernandes, Lauro Sumoy, Oriol Plans, Fernando Arméstar, Cristina Prat

**Affiliations:** 1 Microbiology Department, Hospital Universitari Germans Trias i Pujol, Institut d’Investigació en Ciències de la Salut Germans Trias i Pujol, Universitat Autònoma de Barcelona, 08916 Badalona, Spain; alacoma@igtp.cat (A.L.); gerardgodoytena@gmail.com (G.G.-T.); meissinergomes@gmail.com (M.G.-F.); 2 CIBER Enfermedades Respiratorias, CIBER, Instituto de Salud Carlos III, 08916 Badalona, Spain; 3 Department of Biology and Biochemistry, University of Bath, Bath BA2 7AY, UK; ml418@bath.ac.uk; 4 High Content Genomics and Bioinformatics Unit, Institut d’Investigació en Ciències de la Salut Germans Trias i Pujol, 08916 Badalona, Spain; jsanchez@igtp.cat (J.F.S.-H.); lsumoy@igtp.cat (L.S.); 5 Nuffield Department of Medicine, University of Oxford, John Radcliffe Hospital, Oxford OX3 9DU, UK; bernadette.young@ndm.ox.ac.uk; 6 Intensive Care Unit, Hospital Universitari Germans Trias i Pujol, Institut Germans Trias i Pujol, Universitat Autònoma de Barcelona, 08916 Badalona, Spain; oriolplans@hotmail.com (O.P.); farmestarrodriguez@gmail.com (F.A.); 7 Julius Center for Health Sciences and Primary Care, University Medical Center Utrecht, Utrecht University, 3584 CG Utrecht, The Netherlands

**Keywords:** *Staphylococcus aureus*, mechanical ventilation, pneumonia, toxicity, adhesion, persistence

## Abstract

*Staphylococcus aureus* is a commensal and frequent colonizer of the upper respiratory tract. When mechanical ventilation disrupts natural defenses, *S. aureus* is frequently isolated from the lower airways, but distinguishing between colonization and infection is difficult. The objectives of this study were (1) to investigate the bacterial genome sequence in consecutive isolates in order to identify changes related to the pathological adaptation to the lower respiratory tract and (2) to explore the relationship between specific phenotypic and genotypic features with the patient’s study group, persistence of the clinical isolate and clinical outcome. A set of 94 clinical isolates were selected and corresponded to 34 patients that were classified as having pneumonia (10), tracheobronchitis (11) and bronchial colonization (13). Clinical strains were phenotypically characterized by conventional identification and susceptibility testing methods. Isolates underwent whole genome sequencing using Illumina HiSeq4000. Genotypic characterization was performed with an in-house pipeline (BacterialTyper). Genomic variation arising within-host was determined by comparing mapped sequences and de novo assemblies. Virulence factors important in staphylococcal colonization and infection were characterized using previously established functional assays. (1) Toxin production was assessed using a THP-1 cytotoxicity assay, which reports on the gross cytotoxicity of individual isolates. In addition, we investigated the expression of the major virulence factor, alpha-toxin (Hla) by Western blot. (2) Adhesion to the important extracellular matrix molecule, fibronectin, was determined using a standardized microtitre plate assay. Finally, invasion experiments using THP-1 and A539 cell lines and selected clinical strains were also performed. Repeated isolation of *S. aureus* from endotracheal aspirate usually reflects persistence of the same strain. Within-host variation is detectable in this setting, but it shows no evidence of pathological adaptation related to virulence, resistance or niche adaptations. Cytotoxicity was variable among isolates with 14 strains showing no cytotoxicity, with these latter presenting an unaltered Fn binding capacity. No changes on cytotoxicity were reported when comparing study groups. Fn binding capacity was reported for almost all strains, with the exception of two strains that presented the lowest values. Strains isolated from patients with pneumonia presented a lower capacity of adhesion in comparison to those isolated during tracheobronchitis (*p* = 0.002). Hla was detected in 71 strains (75.5%), with most of the producer strains in pneumonia and bronchial colonization group (*p* = 0.06). In our cohort, Hla expression (presence or absence) in sequential isolates was usually preserved (70%) although in seven cases the expression varied over time. No relationship was found between low cytotoxicity and intracellular persistence in invasion experiments. In our study population, persistent *S. aureus* isolation from airways in ventilated patients does not reflect pathological adaptation. There is an important diversity of sequence types. Cytotoxicity is variable among strains, but no association with study groups was found, whereas isolates from patients with pneumonia had lower adhesion capability. Favorable clinical outcome correlated with increased bacterial adhesion in vitro. Most of the strains isolated from the lower airways were Hla producers and no correlation with an adverse outcome was reported. The identification of microbial factors that contribute to virulence is relevant to optimize patient management during lower respiratory tract infections.

## 1. Introduction

*Staphylococcus aureus* is an important human pathogen in both community and hospital settings. Infections can involve any organ system and can range from asymptomatic colonization to virulent forms of septicemia reflecting its versatility to survive in diverse human niches [1,2,3]. Associations between *S. aureus* colonization and staphylococcal disease have been demonstrated. Lungs represent the major site of infection in the intensive care unit (ICU) and *S. aureus* is one of the most frequent pathogens identified [4], particularly within the first week of mechanical ventilation (MV). Studies of prevalence are based on positive cultures from respiratory samples. However, even when clinicians are confident *S. aureus* has been isolated from respiratory sites, the clinical significance of this remains unclear [5]. Clinicians frequently face the dilemma of distinguishing between microbes colonizing the upper respiratory tract or actively causing airway and/or parenchymal infection [6]. In a recent prospective international study involving 1933 ICU patients from 30 European ICUs, *S. aureus* ICU pneumonia incidence was 4.9 per 1000 ICU patient-days for patients on MV at ICU admission (or shortly thereafter). Patients colonized with *S. aureus* at the time of ICU admission had an almost four-fold higher risk of developing *S. aureus* ICU pneumonia and colonization status was the only independent risk factor [7]. *S. aureus* has been reported to be particularly difficult to eradicate from the respiratory tract in some situations [8]. In a previous study, we showed persistent isolation despite antimicrobial treatment adjusted to susceptibility testing was frequent, even in cases with isolation of methicillin susceptible isolates with no impact on clinical outcome [9]. The repeated and consecutive isolation of *S. aureus* in the lower respiratory tract suggests that the microorganism can survive in this particular niche. Indeed in patients with chronic respiratory diseases such as cystic fibrosis, this mechanism has been widely reported and investigated [10,11]. However, its impact during acute lower respiratory tract infection has not been extensively investigated. Experimental in-vitro approaches characterizing this persistence mechanism using non-phagocytic and phagocytic cell lines [12], including alveolar macrophages, which represent the main components of the first line of defense during lung infections have been developed [13].

*S. aureus* has multiple virulence factors, including surface proteins and secreted toxins whose expression relies on a complex network of regulatory systems [14,15,16]. Fibronectin binding proteins (FnBPs) are members of the microbial surface components recognizing adhesive matrix molecules (MSCRAMMs) that have been implicated in the pathogenesis of staphylococcal infections. *S. aureus* has two FnBPs, FnBPA and FnBPB, both of which play a critical role in surface attachment and adhesion to multiple host cells and tissues [16], including adherence to fibronectin coated surfaces [17,18]. Toxicity has also a significant role in the virulence of *S. aureus* [15]. The ability of pore forming toxins [19], such as leukotoxins and alpha toxin [20] to lyse host cells causes local tissue damage, triggers inflammatory response and can facilitate bloodstream dissemination. A special focus has been placed on the role of alpha toxin during staphylococcal pneumonia, and prophylactic and therapeutic strategies based on the use of monoclonal antibodies targeting virulence factors are under investigation [21,22]. Improving our understanding of the role for virulence factors in *S. aureus* pathogenesis is a critical step for successful vaccine development [23].

In the present study we conducted an extensive genotypic and phenotypic characterization of clinical strains of *S. aureus* isolated from patients during mechanical ventilation. The objectives were (1) to investigate bacterial genomes in sequential isolates in order to identify changes related to the pathological adaptation to the lower respiratory tract and (2) to explore the relationship between specific phenotypic and genotypic features with the patient’s study group, persistence of the clinical isolate and clinical outcome.

## 2. Results

### 2.1. Patients and Strains

The collection of bacterial isolates consisted of 94 strains, corresponding to 34 patients that were classified as follows: pneumonia (10 patients), tracheobronchitis (11 patients) and bronchial colonization (13 patients) (Table 1). Among the 34 cases, 24 were considered as persistent isolation in respiratory sample (>7 days). For 12 patients, *S. aureus* was isolated in ETA but also in other clinical samples (blood culture (BC), pleural effusion, nasal swab and/or central venous catheter) during the same episode. See additional details and the main characteristics of the study population, including the number of isolates per patient in Table 1.

Sixty-seven (71.3%) isolates were methicillin susceptible (MSSA) and 27 (28.7%) were methicillin resistant (MRSA). All strains were vancomycin, teicoplanin and daptomycin susceptible, except for one MRSA isolate that was daptomycin resistant.

### 2.2. Genomic Characterization

**Figure 1:**
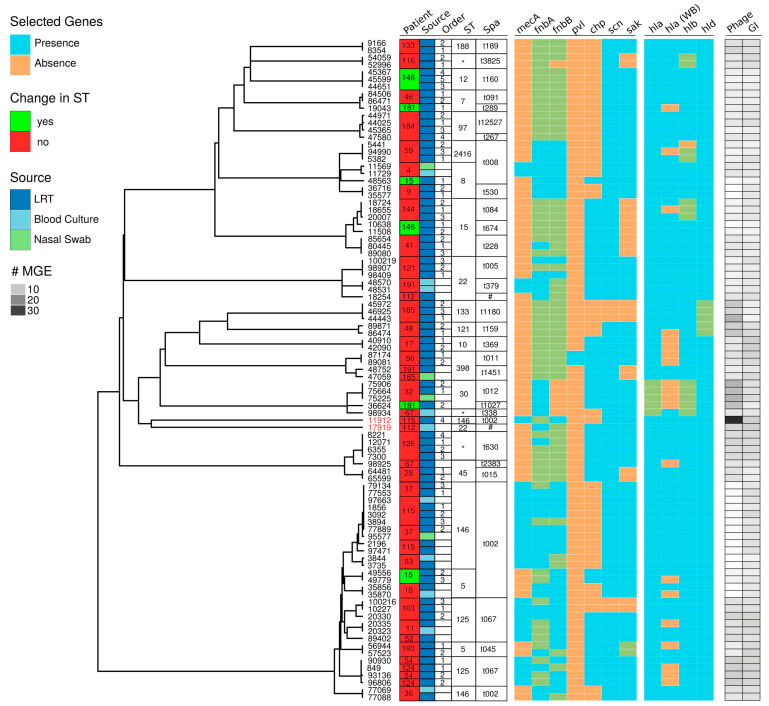
Summary of genomic characterization results for all samples analyzed in this study. From left to right: (1) Sequence similarity clustering tree; (2) bacterial isolate numeric identifiers (culture number in Supplementary Table S2) Red color corresponds to samples containing *Enterococcus faecalis* DNA; (3) patient, individual patient numeric identifiers (ID patient in Supplementary Table S2), color coded by status (red, no change; green, change) diverging sequence type (ST) assignment between isolates from the lower respiratory tract (tracheal aspirate and sputum) of the same patient; patients 15, 146 and 181, are graphically labeled. (4) Origin, isolation source with the following color code: dark blue, lower respiratory tract (LRT) including tracheal aspirate, pleural effusion, protected brush specimen and sputum; light blue, nasal swab and light green, blood culture (including one central venous catheter); (5) order, corresponding to order of isolation for serial LRT samples and (6) ST (strain type) multilocus sequence typing (MLST) assignment results. Asterisks (*) indicate ST could not be assigned. (7) Spa typing protein results. Code corresponds to Ridom Spa Server nomenclature (https://spaserver2.ridom.de/, accessed on 1 August 2020). Asterisks (*) indicate Spa type could not be assigned; hash (#) corresponds to an unknown spa type, see Supplementary Table S2 for details. (8) Genotyping results for several genes of interest, color coded by presence (cyan), absence (salmon) or ambiguous (green). The first block corresponds to some resistance and virulence genes (*mecA*, *fnbA*, *fnbB*, *pvl*, *chp*, *scn* and *sak*) and the following block contains hemolysin encoding genes (*hla*, *hlb* and *hld*), including Western blot (WB) results for Hla (Hla WB). (9) MGE, mobile genetic elements. Grey scale quantitative heatmap showing the number of phage insertions (Phage) and genomic islands (GI) detected for each sample. Data from 91 isolates and 34 individuals is shown.

Results of the genomic characterization using BacterialTyper (https://github.com/HCGB-IGTP/BacterialTyper) (v0.4.2, accessed on 1 August 2020) are available in Figure 1 and Supplementary Table S3. Clustering results, multilocus sequence type (MLST), spa typing, number of mobile genetic elements (phages and genomic islands) and the resistance and toxin/virulence factor genes selected are shown. See details and additional information in Supplementary Table S3.

Isolates belonged to 16 MLST. The most dominant MLST types (considering the 94 isolates) identified were ST146 (17.8%), ST125 (12%), ST15 (9.5%), ST 8 (8.3%) and ST 22 (8.3%). Seven strains could not be assigned to any known sequence type (ST) (allele not described for glpF (4 isolates), for pta (2 isolates) and for yqiL (1 isolate)). Interestingly, for three cases (ID 15, 146 and 181) more than one ST was identified per patient. For ID 15, ST 8 and ST 5 were detected; ID 146, ST 12 and ST15; and ID 181, ST7 and ST30. For ID146 and 181, ST were clustered in two different clinical episodes, while for the remaining one (ID15), both ST were simultaneously detected during the same episode. For the following ID cases (ID 36, 37, 41, 59, 115, 126 and 193), although strains presented the same ST (considering each patient individually), the gene profile displayed some differences (presence/absence of a selected gene) (Figure 1).

A total of 29 different spa types were identified following the Ridom Spa Server nomenclature (https://spaserver2.ridom.de/, accessed on 1 August 2020). Spatypes t002 (20.21%), t067 (10.63%) and t008 (6.38%) respectively, were the most common repeat types identified. Other repeat types identified were always under 5% frequencies. One spa repeat, which was found in two isolates from a single patient, corresponded to an unknown spa type (26–23–13–23–36–16–28).

For two strains (patient ID 112 and 115) (culture numbers 17919 and 11912), the pipeline identified the presence of DNA from *Enterococcus faecalis*, based on assembly size, quality control and strain identification. For one of the samples, blood culture was positive for both microorganisms. For the other sample that was a pleural effusion culture, it might correspond to a cross contamination.

Finally, three isolates (from the same patient, ID 103) were identified using BacterialTyper as *Staphylococcus pseudointermedius*. Additionally, neither MLST nor spa typification resulted positive for these samples. Reanalysis of these samples using MALDI-TOF (Bruker) confirmed them as *S. pseudointermedius*.

### 2.3. Within-Host Variation

**Figure 2:**
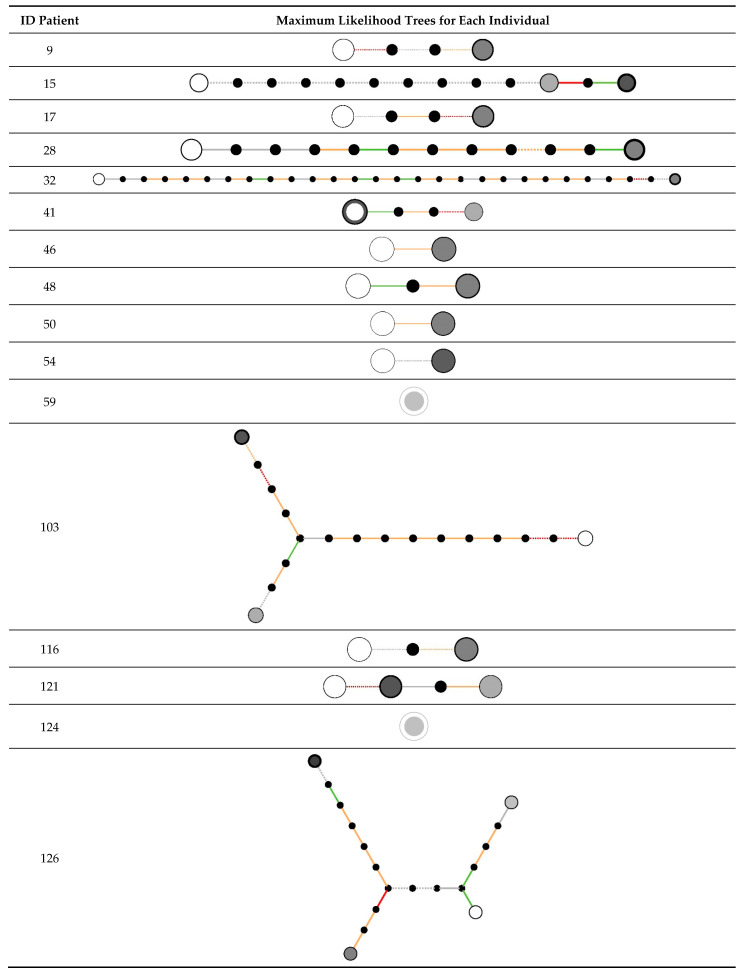

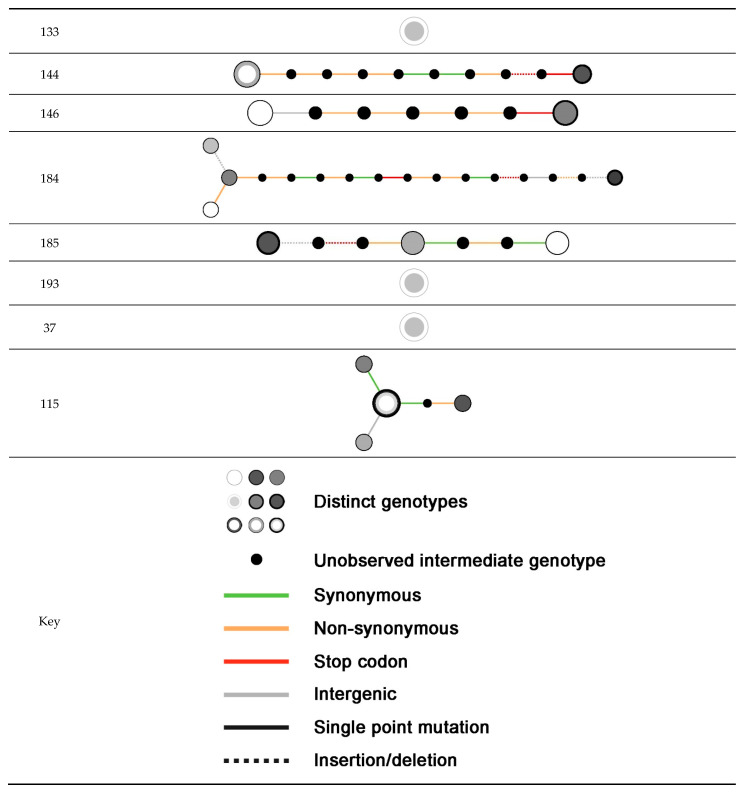
Maximum likelihood trees of the populations found from each individual. Circles represent distinct genotypes, and are shaded according to the time of isolation of the genotype: white is earliest, darkest grey is latest. Branches between nodes are shaded according to the types of variants found: intergenic (grey), synonymous (green), nonsynonymous (orange) and nonsense/premature codon stop (red). SNPs and indels are represented by solid and dashed lines, respectively. Small black circles represent hypothetical intermediate genotypes. Data from 25 individuals is shown.

For this analysis, we selected 73 clinical strains, corresponding to 25 patients with between 2 and 6 isolates from the lower respiratory tract. In 23 out of 25 individuals, 2 or more sequential isolates were closely related. In 21 out of 25 patients, all isolates from the same patient were closely related, with strain replacement seen in the remaining four individuals (Table 2). Maximum likelihood trees of the populations found from each individual are shown in Figure 2. Related isolates were highly similar: in 5/23, no genetic variation was detected, with 18/23 showing at least one genetic variant. Up to 31 within-host variants were detected (median 3 variants, IQR 1–8) with a total of 134 variants identified (Supplementary Table S4). The majority of variants were non-synonymous or protein truncating. No variations were identified in the accessory gene regulator locus or in any cell wall anchored proteins. Gene set enrichment analysis showed no clustering of variants by gene, in any sets of genes with a known shared ontology or within sets of genes with shared regulation.

For the evaluation of cytotoxicity and adhesion capacity only the first isolate from ETA sample was considered, as within-host analysis results showed that repeated isolation of *S. aureus* from ETA frequently reflected the persistence of the same strain. Strains isolated from the other anatomical locations were also included. Cytotoxicity and Fn adhesion results for the 48 tested strains are shown in Supplementary Figure S1.

### 2.4. THP-1 Cytotoxicity

**Figure 3:**
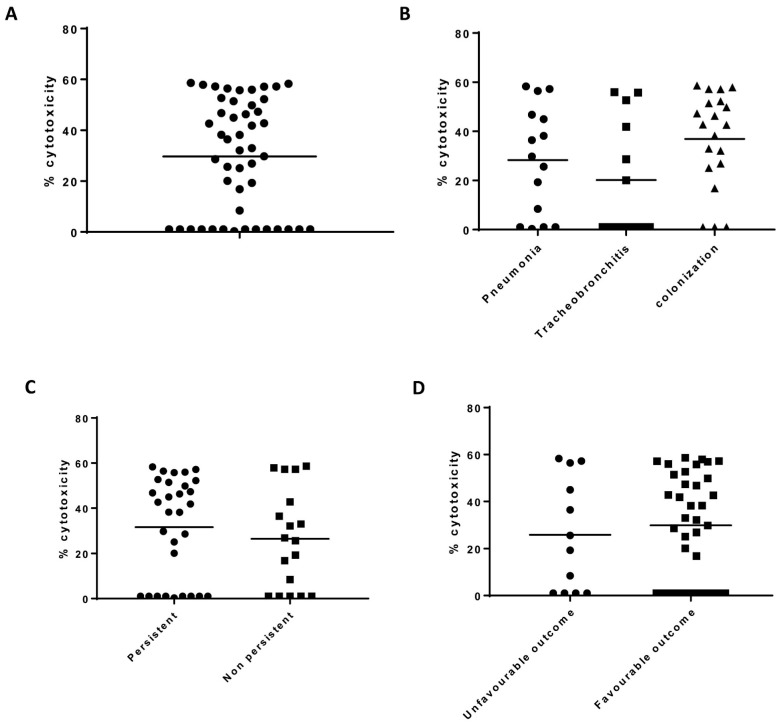
Cytotoxicity profile for the 48 clinical strains. (**A**). Distribution of cytotoxicity results. (**B**). Cytotoxicity according to the study group considered. (**C**). Cytotoxicity according to persistent isolation in the respiratory tract. (**D**). Cytotoxicity according to the clinical outcome, defined as development of respiratory complications and/or mortality related. Mann–Whitney U test and one-way ANOVA test were used. No statistical differences were found.

Cytotoxicity levels were variable among isolates and ranged between 1% and 60% (Figure 3). For USA300 strain, the cytotoxicity value was 50%. Fourteen isolates showed no cytotoxicity. These corresponded to nine patients (Patient ID 11, 50, 67, 83, 103, 112, 115, 144 and 181), 5 were diagnosed as tracheobronchitis, 2 as pneumonia and 2 as bronchial colonized. Isolates belonged to seven different ST; 10 were alphatoxin producers and 4 non producers and one was *pvl* positive. Two patients had an unfavorable outcome. Globally, no statistical differences were reported according to the study group, persistence nor clinical outcome (Figure 3).

### 2.5. Fn Binding Capacity

**Figure 4:**
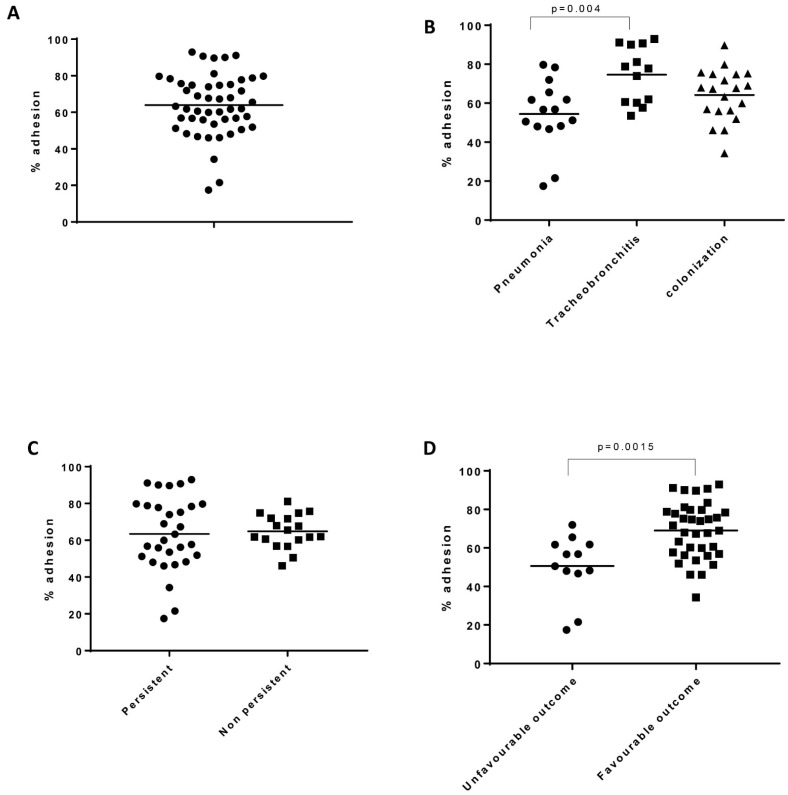
Adhesion profile for the 48 clinical strains. (**A**). Distribution of adhesion results. (**B**). Adhesion according to the study group considered. (**C**). Adhesion according to persistent isolation in the respiratory tract. (**D**). Adhesion according to the clinical outcome, defined as development of respiratory complications and/or mortality related. Mann–Whitney U test and one-way ANOVA test were used. Statistical differences were found when comparing values according to the study group and the clinical outcome.

Adherence was variable among the 48 isolates as shown in Figure 4, although most of the strains had values between 50% and 80%. Two strains presented the lowest values (<20%), and both were isolated from the same patient that belonged to the pneumonia group (Patient ID 4). Both strains were positive for *fnbA* and *fnbB*. For wild type strain (8325.4), adherence corresponded to 100% and the value obtained for 8325.4 Δfnb (deletion of both FnBPA and FnBPB) was 54%. Strains isolated from patients with pneumonia presented a lower capacity of adhesion in comparison to those isolated during tracheobronchitis (*p* = 0.004). Regarding persistence, no statistical differences were reported. Adhesion values reported for isolates from respiratory complications and/or the mortality related group were significantly lower in comparison to isolates from favorable outcome cases (*p* = 0.0015). Regarding the frequency of strains according to the presence of fnb genes, 85 strains were positive for *fnbA* and *fnbB* (90.4%), 6 strains were positive for fnbA/negative for *fnbB* (6.4%) and 3 strains were negative for both genes (3.2%). The latter ones corresponded to *S. pseudointermedius* isolates. In this case, ambiguous results for the genomic characterization of *fnbA*/*fnbB* genes were due to accumulation of non-synonymous variants or partial assemblies as a result of misassembles in the repeated regions of these proteins [24].

### 2.6. Alpha Toxin Detection by Western Blot

**Figure 5:**
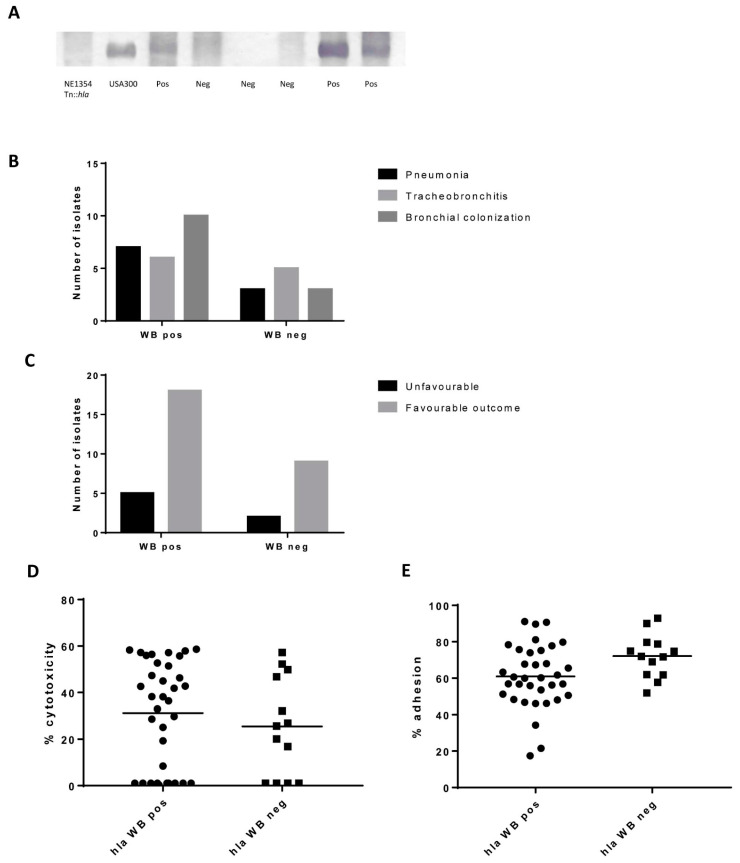
Hla expression. (**A**). Image with examples of positive (pos) and negative (neg) Hla WB results, including clinical isolates and control strains (USA300, NE1354 Tn::*hla*). (**B**). Number of isolates (one per patient) according to the study group and Hla expression (positive/ negative). (**C**). Number of isolates (one per patient) according to the clinical outcome and Hla expression. (**D**). Cytotoxicity and (**E**). Adhesion values according to Hla expression. Mann–Whitney U test and Fisher’s exact test were used. No statistical differences were found.

Given the fact that Hla is one of the most studied toxins involved in airway infection, we were interested in analyzing its expression in the complete set of 94 strains. Eighty-six isolates (94.5%) had the *hla* gene present. Globally, Hla expression by western blot (WB) was detected in 71 isolates (78%). Examples of this WB are shown in Figure 5A. For five strains, the *hla* gene was reported as ambiguous, as it encoded a truncated form of the protein (amino acid position 112/319). However, one of these strains (culture number 98934) had a positive WB. Considering one strain per patient (the first one isolated from the lower respiratory tract), the distribution of Hla results according to the study group is shown in Figure 5B (*p* = 0.496). The distribution of Hla results according to the outcome are shown in Figure 5C (*p* = 0.999). There was no statistical significance between Hla expression and cytotoxicity and adhesion values (Figure 5D,E).

For the analysis of Hla expression in consecutive isolates, we selected 65 clinical strains that corresponded to 23 patients classified as persistent isolation. There is one case (Patient ID 4) that was considered persistent but isolates available for genotypic and phenotypic characterization were a nasal swab and a blood culture. For 16 cases, there were no changes in Hla expression: in 12 cases all isolates were Hla positive (Patient ID 9, 28, 41, 46, 103, 116, 121, 126, 133, 146, 184 and 185) and for 5 cases all isolates were Hla negative (Patient ID 17, 32, 50, 124 and 181). For two cases (Patient ID 15 and 54), Hla was initially detected but later no expression was reported, and in two cases (Patient ID 48, 59, 144 and 193), Hla was initially absent but later reported. Overall, Hla expression (presence or absence) in sequential isolates was usually preserved (72.8%) although in six cases the expression varied over time. Among these six cases, in five of them within-host analysis showed acquisition of a novel strain over time.

### 2.7. Invasion and Persistence Assay

**Figure 6:**
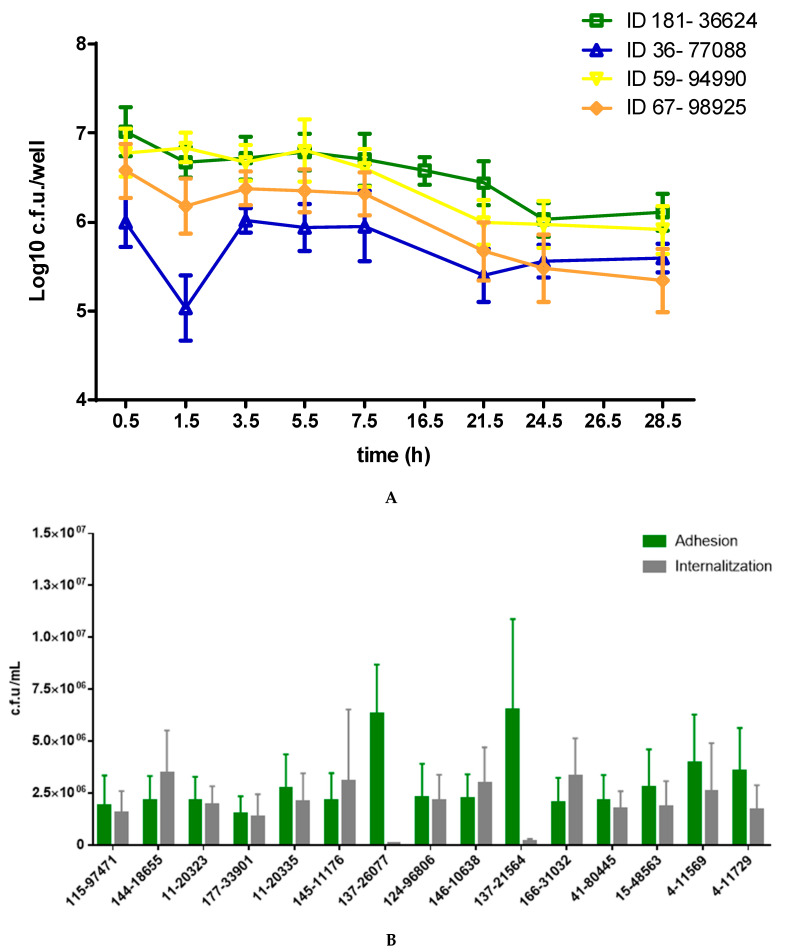
Invasion assays in murine alveolar macrophage (MH-S) and A549 cell lines. (**A**). Time course analysis for 4 *S. aureus* clinical isolates in infected MH-S. Log 10 c.f.u./well are the average of three independent experiments. (**B**). Adhesion and internalization bacterial counts after infection of the A549 cell line with selected clinical strains. c.f.u./mL are the average of three independent experiments.

Regarding the murine alveolar macrophage (MH-S) invasion assay, all isolates showed a similar dynamic, regardless of the clinical group, persistence and cytotoxicity (Figure 6A). For tree isolates, there was an initial decrease in bacterial counts after the 1 h incubation with gentamycin and a slight increase in the following 2 h incubation. Bacterial counts remained constant until 7.5 h post infection (p.i). From that time point a slight decrease was reported. From 21.5 to 28.5 h p.i, counts remained constant, suggesting a balance between bacterial killing and intracellular survival. Regarding A549 assay (Figure 6B), bacterial counts showed that all strains adhered efficiently and are internalized after 2 h, regardless of the cytotoxicity (addressed by THP-1 cytotoxicity assay). Strains are ordered in the x-axis according to the cytotoxicity value obtained. Interestingly, there were two strains from the same patient (ID 137) that reported the highest counts for adhesion time point (26077 and 21564), whereas no counts were detected for the internalization time point.

## 3. Discussion

*S. aureus* pathogenesis during bacteremia, endocarditis and skin and soft tissue infections has been extensively evaluated [1]. Genomic signatures in *S. aureus* of invasive or persistent infection have been investigated, although with conflicting results [25,26,27]. *S. aureus* clinical features in the respiratory tract can range from asymptomatic carriage to severe invasive disease, demonstrating a switch in virulence regulation [1,28]. Most studies of pneumonia have focused on assessing differences of outcome between MRSA and MSSA, because of its importance regarding available antimicrobial options [29,30] and identifying the potential role of specific virulence factors such as Panton–Valentine leukocidin [31] and alpha-toxin [32] in an adverse clinical outcome. Although *S. aureus* persistent isolation in the lower respiratory tract is distinct from invasive infection, isolates might also be subjected to the pressures of the immune response, lack of nutrients, coexisting microorganisms and antimicrobials [33]. Thus, access to sequential isolates offers an opportunity to characterize pathological host adaptation in this anatomical location. In the current study, we performed an exhaustive genotypic characterization of *S. aureus* clinical strains sequentially isolated from patients under mechanical ventilation [9]. Previous studies of invasive *S. aureus* infection have demonstrated low within-host diversity in infecting populations, and changes in regulatory genes, especially the Agr locus, associated with the development of bacteremia and invasive infections [34,35,36]. In contrast, while within-host variation is detectable in our setting, we found no evidence of pathological adaptation related to virulence, resistance or niche adaptation. There was no observable difference between the phenotypes of colonization and infection in the degree of diversity observed within repeated samplings. Colonization and infection of the lower respiratory tract may involve distinct evolutionary pressures compared with the bloodstream or deep soft tissue. Clinically, repeated isolation of *S. aureus* from the lower respiratory tract did not correlate with worse outcomes compared with patients with only a single *S. aureus* tracheal aspirate culture [9]. As it has been previously mentioned, from a clinical perspective, in some circumstances the diagnosis of respiratory tract infections can be complex [6]. Regarding the three study groups defined, there is no gold standard for clear distinction, and this may be a reason for not seeing differences between study groups in terms of within-host variation.

An interesting aspect of our study is that it represents a non-uniform population of clinically relevant isolates. Cases were retrospectively included on the basis of a *S. aureus* positive respiratory specimen culture result and the storage of the clinical isolate at −80 °C for further characterization. Inclusion was not dependent on patients’ criteria and strains were not collected in the same time points for all patients. Indeed, sequence clustering and ST among isolates was very heterogeneous (Figure 1), highlighting wide genetic diversity and thus low patient-to-patient transmission in the ICU. Cytotoxicity displayed a wide range of values and did not correlate with any of the variables considered (study group, persistence and clinical outcome), including the expression of alpha toxin. For most isolates, values were lower than the one found for the reference strain (USA300), suggesting low toxicity for the respiratory isolates. This low cytotoxicity was found in bronchial colonization and pneumonia isolates, which could be seen as opposite clinical situations, suggesting that virulence factors involved in tissue destruction are not the sole or major determinants of pathogenesis. Low cytotoxicity is considered an adaptation strategy to facilitate intracellular persistence and chronic infections [37]. With the experimental approaches performed, this relationship was not demonstrated, although we showed that *S. aureus* was able to persist intracellularly in two different human cell lines. It is important to remind that patients included in our study did not present a chronic infection, but an acute lower respiratory tract infection, which in some cases lead to persistent bronchial colonization.

The majority of strains were alpha-toxin positive and only two strains were PVL positive. One explanation would be that in order to adapt to the lower respiratory tract *S. aureus* downregulates *agr* expression among others [3]. In addition, for medium/long term survival in the airways, toxin expression would have to be tightly regulated in favor of persistence. In a study by Laabei M et al., a higher propensity for low cytotoxicity isolates to cause bacteremia was reported [38], and this was explained by evolutionary trade-offs and bacterial fitness. Adherence was also variable among respiratory isolates, reflecting heterogeneity within the bacterial population. Isolates from pneumonia group displayed significantly lower values of adhesion in comparison to tracheobronchitis group. Adhesion was also significantly lower for cases with unfavorable outcome. This is consistent with the fact that among the 34 clinical cases, 7 presented development of respiratory complications and/or mortality related. All of them belonged to the pneumonia study group, which includes those patients with worse outcomes [9]. Adherence to fibronectin did not differ between isolates with one or two *fnb* genes, as previously shown [17]. Interestingly, there were 16 strains that lacked both genes, but still showed Fn adhesion capacity. The explanation for this result can be that many cell wall anchored proteins show functional redundancy [16].

Alpha toxin is a relevant virulence factor in *S. aureus* induced pneumonia [20,28] and targets multiple host cell types. Alpha toxin expression depends on a complex interplay of regulatory networks, alternative sigma factors and transcription factors [20] and during infection its production is also modulated in the presence of phenol-soluble modulin peptides [39]. It has been proposed that the *hla* gene evolved together with *S. aureus* genetic background [40]. In the same direction, Tabor et al. found that *hla* was highly conserved among isolates from the lower respiratory tract [32]. In our study, almost all of the isolates were positive for the *hla* gene, and about 75% of them were Hla producers. Particularly for the pneumonia study group, almost 90% of the isolates were Hla producers, suggesting an association with this clinical condition. Although the number of patients in our cohort is low, it provides evidence regarding the role of this virulence factor during lower respiratory tract infections. Regarding sequential isolates, its expression was usually preserved, with the exception of a few cases for which the expression varied over time. Most of these cases, corresponded to the cases identified in within-host analysis as novel strains acquisitions, different than the index case.

The complex interplay between host (host genetic risk factors, epidemiological characteristics and presence of comorbidities) and microbial genetic determinants (arsenal of virulence factors and tight gene expression regulation) is not only dependent on the clinical condition [6,41]. It is important to mention that in-vivo conditions might be different than in-vitro conditions. It is difficult to assess which growth phase is relevant in terms of toxicity and adhesion during in-vivo disease. However, for the different tests performed in the current study, bacterial supernatants were prepared, accordingly, at the early exponential phase for adhesion assays, and at the late exponential to stationary phase of growth for toxicity and alpha toxin secretion assays. It is widely accepted that growth media can influence virulence factor secretion [42] and literature supports TSB as one of the more appropriate to perform phenotypic assays exploring *S. aureus* pathogenesis.

In summary, our results show that there is a diversity of *S. aureus* isolates in terms of CC/ST and virulence/resistance genes profile. Within-host variation is detectable in our setting, but it shows no evidence of pathological adaptation related to virulence, resistance or niche adaptation. Cytotoxicity was variable among strains, but no association with study groups, clinical outcome and persistence was found, whereas isolates from patients with pneumonia and unfavorable outcome had lower Fn adhesion capability. Most of the strains were alpha-toxin producers. Hla expression (presence or absence) in sequential isolates was usually preserved although in a small proportion of the cases, the expression varied over time. The identification of microbial factors that contribute to virulence is relevant to optimize patient management during lower respiratory tract infections.

## 4. Material and Methods

### 4.1. Patients and Strains

The study is observational. Patients admitted to ICU over a 2 year period (2012–2013) and from whom *S. aureus* was isolated in at least one endotracheal aspirate (ETA) were selected as described previously [9]. All patients at inclusion were under mechanical ventilation. Ethical approval was provided by the Institutional Review Board: Comitè d’Ètica de la Investigació de l’Hospital Germans Trias i Pujol. Sample collection followed the standard of care and was based on clinical (and radiological when applicable) suspicion of infection, and not as surveillance culture. No intervention was applied to the collection timing. Epidemiological, radiological, clinical and microbiological data were recorded. Adverse clinical outcome included the development of respiratory complications following definitions from Ferrando et al. [43], including empyema and septic shock and infection-related mortality. Isolation in ETA was defined persistent when it lasted ≥7 days despite antimicrobial treatment adjusted to susceptibility profile, and was considered regardless of the study group. Patients were clustered into pneumonia, tracheobronchitis or bronchial colonization according to the Clinical Pulmonary Infection Score (CPIS). Pneumonia (CPIS ≥ 6) was considered ventilator associated (VAP) when its onset occurred after ≥48 h of MV. Ventilator associated tracheobronchitis (VAT) was defined when the following 4 criteria were present: fever (≥38 °C) with no other recognizable cause, purulent secretion, positive culture of respiratory specimen at significant threshold, (by semiquantitative culture) [44] and no radiographic signs of new pneumonia [4]. Bronchial colonization was defined as *S. aureus* isolation in respiratory sample but at least one of the aforementioned clinical, microbiological and radiological features absent. Isolates were stored in maintenance freeze medium (15% glycerol) (Oxoid, TP15731) at −80 °C until use.

### 4.2. Whole Genome Sequencing

*S. aureus* isolates were grown overnight in 2 mL of tryptic-soy broth (TSB). Overnight cultures were centrifuged at 5000× *g*, 5 min and supernatants were discarded. Bacterial pellet was suspended with enzyme solution (200 μg/mL lysostaphin (AMBI products, Lawrence, KS, USA); 20 mM Tris·HCl, pH 8.0; 2 mM EDTA; 1.2% Triton X-100) and incubated for 1 h at 37 °C. Incubation with 4 μL RNAase A (stock solution 100 mg/mL) (Qiagen 19101) (Qiagen, Hilden, Germany) was also performed (1 h at 56 °C). Genomic DNA (gDNA) was prepared using DNA mini and Blood mini kit (Qiagen 51304) (Qiagen, Hilden, Germany) according to the manufacturer’s instructions. gDNA concentration and integrity was checked with picogreen fluorometry and SYBR-green agarose gel electrophoresis, respectively. A minimum of 1 μg DNA resuspended in AE buffer (30 ng/mL) was submitted to the Wellcome Trust Centre for Human Genetics (WTCHG) (University of Oxford, Oxford, UK) and subjected to genome sequencing on an Illumina HiSeq4000, with 2 × 150 bp long paired end reads. The whole-genome shotgun project has been deposited in DDBJ/ENA/GenBank under accession number PRJNA673063. This project repository includes raw data, sample details and sequencing libraries information (Supplementary Table S1).

### 4.3. Genomic Characterization

We generated a genomic characterization for all samples using the in-house pipeline named BacterialTyper [45] (https://github.com/HCGB-IGTP/BacterialTyper, accessed on 1 August 2020). It is written in Python with a modular architecture and based on an open-source software and databases engines. Multiple tasks are performed by each module that can integrate multiple software packages. For this analysis we used modules regarding: generation of raw data quality control (FastQC [46] and MultiQC) [47]); assembly (Spades [48]), annotation (prokka [49]) and quality control (BUSCO [50]); multilocus sequence typing (MLST) (MLSTar [51]) and staphylococcal protein A (spa) characterization (spaTyper [50,52]); bacterial strain identification (kma [53]); analysis of putative pathogenicity islands (islandpath [54]) and phage insertions regions (Phispy [55]); sequence similarity clustering (mash [56,57]); generation of a virulence and resistance profile (ARIBA [58]) based on external databases such as the Virulence Factor Database (VFDB [59]) and Comprehensive Antibiotic Resistance Database (CARD [60]). For this analysis results might be either presence, absence or ambiguous. We set as ambiguous any result that is not clearly determined to be present/absent by ARIBA. We would include in this category partial genes (<80% assembly length) or these genes present but not producing the phenotype (e.g., gene is truncated or contains frameshift variants, etc.).

Genomic characterization results were summarized using R packages treeio [61], ggtree and ggtreeExtra [62]. We included: sequence similarity clustering tree information; selected genes from the resistance and virulence profiles; MLST, spa typing results and patient or phenotypic information associated for each sample analyzed.

### 4.4. Within-Host Variation Analysis

For each sample, reads were mapped to a reference strain (MRSA252) [63] using Stampy v 1.0.22 [64]. Repetitive regions identified using BLAST [65] were masked prior to variant calling, and bases at each position were called using previously described quality filters [63,64,65]. Reads were assembled de novo into contigs using Velvet v1.0.18 [66] and using Velvet Optimiser v2.1.7 [66] to choose kmer lengths for each assembly. MLST was determined by identifying MLST alleles in assemblies with BLAST, and comparison to an online database at http://saureus.mlst.net (accessed on 1 August 2020). For isolates of the same sequence type (ST) obtained from a single individual, we identified all variants within the host population using Cortex [67] to identify SNPs and indels. SNP distances were used for comparison between groups. Variants were annotated for their location and predicted effect on protein by comparison to reference genomes using BLAST. Gene set enrichment analysis was performed as previously described [35], testing for genes (and sets of genes with a shared ontology or shared regulatory control) with a higher than expected number of variants.

### 4.5. Phenotypic Characterization

Clinical strains were phenotypically characterized by conventional identification and susceptibility testing methods, interpreted according to the Clinical and Laboratory Standards Institute. Antimicrobials tested were: penicillin, oxacillin, erythromycin, clindamycin, cefoxitin, cotrimoxazole (trimethroprim/sulfamethoxazole), gentamicin, rifampicin, ciprofloxacin, vancomycin and mupirocin. In addition, minimal inhibitory concentration (MIC) for vancomycin, daptomycin and teicoplanin were determined with UMIC commercial assay (Biocentric, Hain Lifescience, Germany). Reference strains used were Newman, USA300, 8325.4, 8325.4 Δfnb and NE1354 Tn::*hla* WB.

### 4.6. THP-1 Cytotoxicity Assay

To investigate the gross cytolytic activity of the isolates collection, the toxicity of bacterial supernatants to the immortalized monocyte–macrophage THP-1 cell line was evaluated using an adapted protocol already published [36,68]. *S. aureus* isolates were grown overnight in 1.5 mL of TSB during 15–18 h at 37 °C, 180 rpm. Overnight cultures were centrifuged at 4000 rpm, 10 min and supernatants filtered through 0.2-micron filter. The monocyte macrophage THP-1 cell line was grown in RPMI-1640, supplemented with 10% heat-inactivated fetal bovine serum, Hepes 10 mM and antibiotics/antifungals at 37 °C in a humidified incubator with 5% CO_2_. Cells were routinely viewed microscopically every 48–60 h and harvested by centrifugation at 1200 rpm for 5 min at room temperature and resuspended to a final density of 2–4 × 10^5^ cells/mL. To monitor *S. aureus* toxicity, 45 μL of cells (1.7 × 10^5^ cells/mL) were incubated with 45 μL of bacterial supernatant and incubated for 12 min at 37 °C with 5% CO_2_. Cell viability was quantified using Presto Blue (Invitrogen) according to the manufacturer’s instructions. Experiments were done in triplicate and repeated three times. Error bars indicate ±95% confidence interval.

### 4.7. Fibronectin Binding Assay

To investigate fibronectin binding protein adherence to human fibronectin, an adapted protocol previously published was used [18]. *S. aureus* isolates were grown overnight in 5 mL of tryptic soy broth (TSB) during 15–18 h at 37 °C, 180 rpm. A subculture (1/200) was incubated for 2 h at 37 °C, 180 rpm and later centrifuged at 3000× *g* during 10 min. The adhesion of bacteria to human fibronectin (Fn) was assessed in a microtitre plate assay. Fn (10 µg/mL in 100 µL of PBS) was immobilized onto flat-bottomed microtitre (Nunc Maxisorp, Thermo Fisher Scientific, Waltham, MA, USA) plates by incubation at 4 °C for 18 h. Wells were washed once and blocked with 200 µL of 3% BSA in PBS at 25 °C for 1 h. Blocked wells were washed with PBS and incubated with 100 µL of bacterial suspension (1 × 10^8^ CFU/well) at 37 °C for 1 h. Non-adherent bacteria were removed by washing with PBS and adherent bacteria were fixed with 2.5% paraformaldehyde (5 min, 25 °C). Fixed bacteria were stained with 100% crystal violet (5 min, 25 °C). Two washing steps with PBS were done before solubilization of crystal violet with 100 μL 7% acetic acid (5 min, 25 °C). The contents of each well were quantified by measurement at Abs 595 nm using a microplate reader (Varioskan, Thermofisher Scientific, Waltham, MA, USA). Experiments were done in triplicate and repeated three times. Error bars indicate ± 95% confidence interval.

### 4.8. Detection of Secreted Hla

Cultures of *S. aureus* strains were grown overnight in TSB (180 rpm, 37 °C) and cultures were normalized to an OD600 nm = 2 before supernatant precipitation [69]. Bacteria were sedimented by centrifugation (14,600 rpm, 10 min) and the supernatant was incubated and precipitated with trichloroacetic acid (14,600 rpm, 20 min, 4 °C). Following three acetone washes, protein precipitants were resuspended in urea (8 M)-NaOH (1 M) solution and separated on a 14% SDS-PAGE gel. For each tested strain, the same volume of protein precipitants solution (40 μL sample and 20 μL loading buffer) was loaded onto each gel. Hla was detected by Western blotting using a 1:3000 dilution of a rabbit polyclonal antibody raised against Hla (S7531, Sigma, St. Luis, MO, USA) for 1 h at room temperature. Immunoblots were washed and incubated with horseradish peroxidase-coupled protein G (1:1000; Invitrogen, Carlsbad, CA, USA) for 1 h at room temperature. Proteins were detected by using the Opti-4CN detection kit (Bio-Rad, Hercules, CA, USA). Strains were categorized as producer or non-producer. Each immunoblot had a positive control that corresponded to protein precipitants from USA300 isolate. NE1354 Tn::*hla* was also included as a negative control.

### 4.9. Invasion Assay

Bacterial strains were selected from the collection (Supplementary Table S2). *S. aureus* strains were streaked onto tryptic soy agar (TSA) and incubated for 18 h at 37 °C, 180 rpm. A subculture dilution (1:200) was prepared and cultured for 2.5 h (37 °C, 180 rpm). After that, bacterial cultures were centrifuged (3000× *g*, 10 min) and pellet resuspended at OD600 nM = 0.9. The murine alveolar macrophage (MH-S) cell line (ATCC CRL-2019) and human epithelial lung cell line A549 (ATCC CCL-185) were cultured according to the manufacturer’s protocol. The MH-S invasion assay was performed as described previously [13]. Cells were infected for 30 min (MOI 25:1). Time course analysis of intracellular *S. aureus* within MH-S was characterized up to 28 h post-infection. Regarding A549 cell adhesion (1 h post-infection) and internalization assay (2 h post-infection), an adapted protocol from [13] was used. Cells were infected for 1 h at MOI 10:1. After 1 h incubation, wells were washed and cells were incubated for 1 h with gentamycin (200 μg/mL) for one hour. At interest time points, 1 h and 2 h post-infection, wells were washed in PBS and lysed with saponin (0.025%). c.f.u were enumerated through serial dilution and plating onto TSB plates and incubated at 37 °C for 24 h.

### 4.10. Statistical Analysis

Continuous variables are expressed as mean and standard deviation, and univariate comparisons were made with Mann–Whitney U test. Multiple groups were compared by an ordinary one-way ANOVA test. Categorical parameters are expressed as absolute numbers and percentage and univariate comparisons were performed with Fisher’s exact test. Differences were considered statistically significant when a *p*-value was <0.05. For gene set enrichment analysis (GSEA), multiple testing was accounted for by adjusting for the numbers of tests performed using a Bonferroni correction. This gave adjusted significance thresholds of 10^−5.2^ for genes, 10^−4.5^ for shared gene ontologies and 10^−4.2^ for shared expression pathways. GSEA was performed using R version 3.3. Additional statistical analysis was performed by using GraphPad Prism version 7.00 (San Diego, CA, USA).

## Figures and Tables

**Table 1:** Description of study population: study group according to clinical criteria, number of isolates, days between isolation, clinical samples collected, persistent isolation in respiratory sample and clinical outcome.

Patient ID	Study Group	Number of Isolates	Clinical Samples ^a^	Persistent Isolation ^b^ (Days)	Respiratory Complications and/or Mortality Related
4	Pneumonia	2	Nasal swab, blood culture	Yes (54) ^c^	Yes
9	Bronchial colonization	2	Tracheal aspirate	No	No
11	Tracheobronchitis	2	Tracheal aspirate, blood culture	No	No
15	Tracheobronchitis	3	Tracheal aspirate	Yes (11)	No
17	Bronchial colonization	2	Tracheal aspirate	Yes (7)	No
18	Bronchial colonization	2	Tracheal aspirate, blood culture	No	No
28	Tracheobronchitis	2	Tracheal aspirate	Yes (8)	No
32	Bronchial colonization	3	Nasal swab, sputum, tracheal aspirate	No	No
36	Pneumonia	2	Tracheal aspirate, blood culture	No	Yes
37	Bronchial colonization	4	Tracheal aspirate (3), nasal swab	Yes (52)	No
41	Bronchial colonization	3	Tracheal aspirate (2), sputum	Yes (57)	No
46	Tracheobronchitis	2	Tracheal aspirate (2)	Yes (8)	No
48	Bronchial colonization	2	Tracheal aspirate (2)	Yes (15)	No
50	Tracheobronchitis	2	Tracheal aspirate (2)	Yes (9)	No
52	Bronchial colonization	1	Tracheal aspirate	No	No
54	Tracheobronchitis	2	Tracheal aspirate (2)	Yes (14)	No
59	Pneumonia	3	Tracheal aspirate (3)	Yes (62)	No
67	Pneumonia	2	Tracheal aspirate, blood culture	No	Yes
83	Bronchial colonization	2	Tracheal aspirate, blood culture	Yes (73)	No
103	Bronchial colonization	6	Sputum (6)	Yes (524)	No
112	Tracheobronchitis	2	Tracheal aspirate, blood culture	No	No
115	Pneumonia	7	Tracheal aspirate (2), blood culture, sputum (2), pleural effusion, protected brush	Yes (74)	Yes
116	Pneumonia	2	Tracheal aspirate	No	Yes
121	Pneumonia	3	Tracheal aspirate	Yes (8)	No
124	Tracheobronchitis	2	Tracheal aspirate	Yes (21)	No
126	Pneumonia	4	Tracheal aspirate	Yes (28)	Yes
133	Pneumonia	2	Tracheal aspirate	Yes (11)	No
144	Tracheobronchitis	3	Tracheal aspirate	Yes (12)	No
146	Tracheobronchitis	5	Tracheal aspirate	Yes (176)	No
181	Tracheobronchitis	2	Tracheal aspirate	Yes (92)	No
184	Bronchial colonization	4	Tracheal aspirate	Yes (15)	No
185	Bronchial colonization	4	Tracheal aspirate (3), nasal swab	Yes (10)	No
191	Bronchial colonization	3	Tracheal aspirate, central venous catheter, blood culture	No	No
193	Pneumonia	2	Tracheal aspirate	No	Yes

^a^, Clinical specimens, the numbers correspond to the total count; ^b^, persistent isolation of *S. aureus* in lower respiratory tract samples, the numbers corresponds to the days of persistence. ^c^, Only nasal swab and blood culture isolates were frozen and available for further characterization.

**Table 2:** Number of within-host variations identified when comparing isolates from patients included in the study.

Patient ID	Number of Isolates	Study Group	Related Isolates Identified	MLST (In Silico) of Earliest Isolate	Number of Within-Host Variants Identified
9	2	Bronchial colonization	Yes	8	3
15	3	Tracheobronchitis	No	5	NA ^a^
17	2	Bronchial colonization	Yes	10	3
28	2	Tracheobronchitis	Yes	45	11
32	2	Bronchial colonization	Yes	30	31
41	3	Bronchial colonization	Yes	15	3
46	2	Tracheobronchitis	Yes	7	1
48	2	Bronchial colonization	Yes	121	2
50	2	Tracheobronchitis	Yes	398	1
54	2	Tracheobronchitis	Yes	125	1
59	3	Pneumonia	Yes	NF	0
103	6	Bronchial colonization	Yes	125	17
116	2	Pneumonia	Yes	NF	2
121	3	Pneumonia	Yes	22	3
124	2	Tracheobronchitis	Yes	125	0
126	4	Pneumonia	Yes	NF	17
133	2	Pneumonia	Yes	188	0
144	3	Tracheobronchitis	Yes	15	10
146	5	Tracheobronchitis	Yes (first 2 of 5)	12	6
181	2	Tracheobronchitis	No	7	NA
184	4	Bronchial colonization	Yes	97	15
185	4	Bronchial colonization	Yes (first 3 of 4)	133	6
193	2	Pneumonia	Yes	5	0
37	3	Bronchial colonization	Yes	146	0
115	6	Pneumonia	Yes	146	4

NA: within-host variants not identified as initial strain replacement by another sequence type; NF: not found; ^a^, This patient had three isolates. The first isolate was a different ST from the next two.

## Data Availability

The whole-genome shotgun project has been deposited in DDBJ/ENA/GenBank under accession number PRJNA673063. This project repository includes raw data, sample details and sequencing libraries information. The data presented in this study are available in supplementary material.

## Supplementary Materials

The following are available online at https://www.mdpi.com/2072-6651/13/2/122/s1, Figure S1: Cytotoxicity and adhesion ability for the 48 clinical strains, each one represented individually, Table S1: *Staphylococcus aureus* samples indexed in the BioProject PRJNA673063. For each sample, the correspondences between the name and the library ID generated in this study and sequence read archive (SRA) and biosample accession ID generated in the submission to NCBI are provided; Table S2: Description of clinical strains tested in invasion assays (MH-S and A549 cell lines). Table S3: Genotype characterization, including MLST and spa type assignment, presence/absence of selected genes (*mecA*, *fnbA*, *fnbB*, *sea*, *seb*, *sec*, *tst*, *eta*, *etb*, *sak*, *pvl*, *hla*, *chp*, *scn*, *hlb* and *hld*) and MGE (numbers of phages and genomic islands) for the 94 clinical strains. ID patient, culture number and date of isolation are also included. Table S4: Description of variants identified between closely related (same MLST) isolates from individuals. Variants are classified according to their predicted effect on the translated protein as synonymous, non-synonymous, frameshift, non-sense or intergenic.
