# Supplementary figures and images for: Genotypic and Phenotypic Characterization of Staphylococcus aureus Isolates from the Respiratory Tract in Mechanically-Ventilated Patients
Source: Toxins (Basel). 2021 Feb 6;13(2):122. doi: 10.3390/toxins13020122 (PMC7915691; doi:10.3390/toxins13020122)

## Slide 1
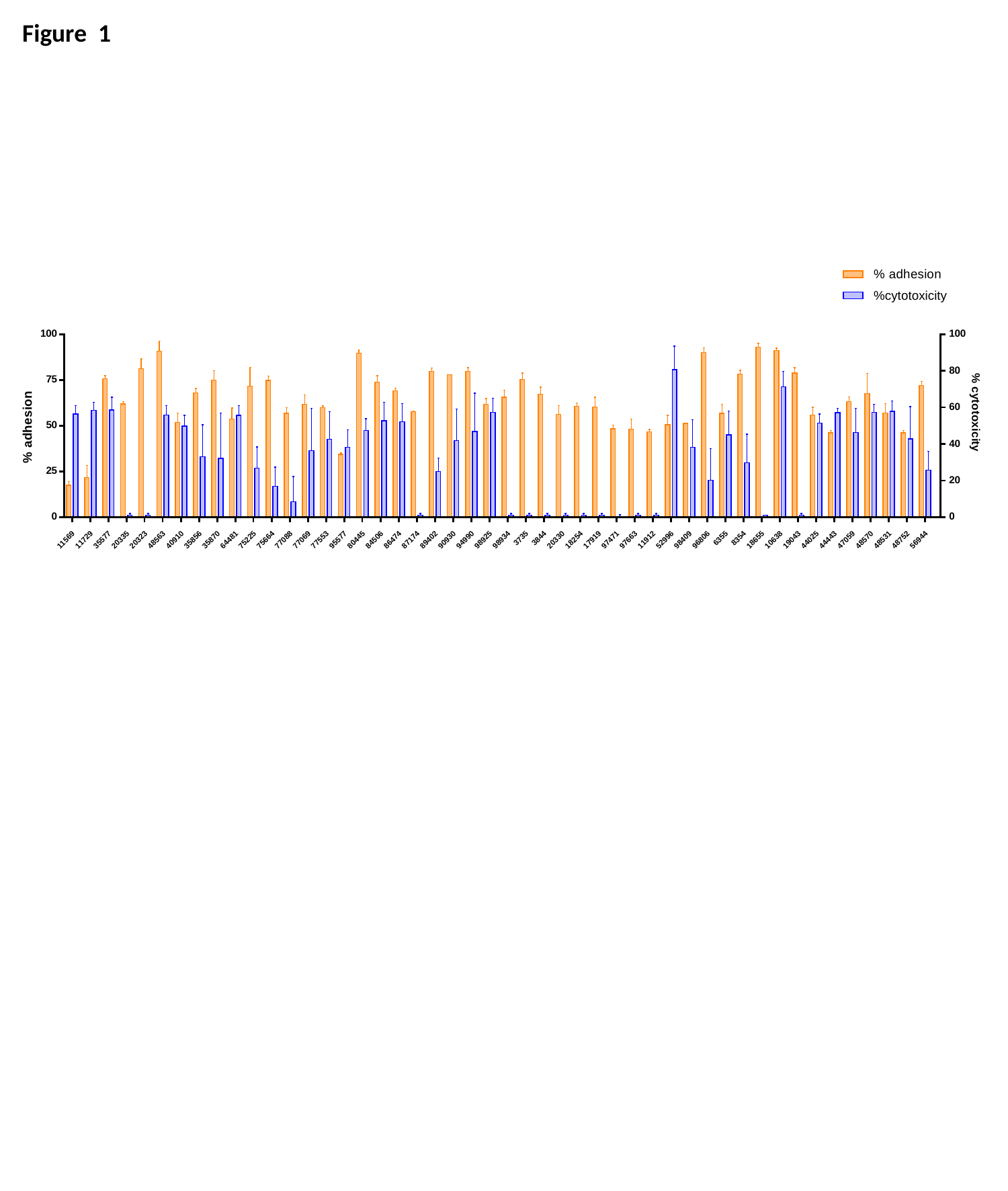

Figure 1

Supplement: Supplementary file 1 [file toxins-13-00122-s001.zip › toxins-1106722-supple-peer/Suppl Figure 1.pptx]
